# Determination of nutritional values of mealworm (*Tenebrio molitor* L.) larvae fed with turmeric (novel food product)

**DOI:** 10.3389/fnut.2026.1812226

**Published:** 2026-04-14

**Authors:** Deniz Aktaran Bala, Sema Sandıkçı Altunatmaz, Filiz Aksu, Cansu Çelik Doğan, İbrahim Akyazı, Dilek Dülger Altıner, Emine Aydın, Seydi Yıkmış, Emad Karrar, Moneera O. Aljobair, Isam A. Mohamed Ahmed

**Affiliations:** 1 Department of Food Processing, Vocational School of Veterinary Medicine, Istanbul University-Cerrahpasa, Avcilar, Istanbul, Türkiye; 2 Department of Physiology, Faculty of Veterinary Medicine, Istanbul University-Cerrahpasa, Avcilar, Istanbul, Türkiye; 3 Department of Gastronomy and Culinary Arts, Tourism Faculty, Kocaeli University, İzmit, Kocaeli, Türkiye; 4 Department of Agricultural Biotechnology, Faculty of Agriculture, Düzce University, Düzce, Türkiye; 5 Department of Food Technology, Tekirdag Namik Kemal University, Tekirdag, Türkiye; 6 Department of Plant Sciences, North Dakota State University, Fargo, ND, United States; 7 Department of Sports Health, College of Sports Sciences and Physical Activity, Princess Nourah Bint Abdulrahman University, Riyadh, Saudi Arabia; 8 Department of Food Sciences and Nutrition, College of Food and Agricultural Sciences, King Saud University, Riyadh, Saudi Arabia

**Keywords:** amino acid profile, *Curcuma longa* (turmeric), functional food, novel food product, *Tenebrio molitor* L.

## Abstract

This study evaluated the nutritional and functional potential of powder obtained from mealworm (*Tenebrio molitor* L.) larvae fed turmeric (*Curcuma longa*) for human consumption. Proximate composition, phenolic fractions (free/bound/total), antioxidant capacity, and bioavailability after *in-vitro* digestion were examined in control (Tm-C) and turmeric-fed group (Tm-T) samples. Phenolics were reported as mg GAE/100 g using the Folin–Ciocalteu method, antioxidant activities were reported as μmol Trolox/g using ABTS•+, CUPRAC, and DPPH• tests; bioavailability was calculated using a two-phase digestion model including pepsin and pancreatin/bile. In the Tm-T group, moisture was 61.77%, protein 24.04%, and fat 6.84%, while in the Tm-C group, these percentages were 64.38, 21.61, and 9.04%, respectively. The dominant fraction of phenolic compounds was bound phenolics, ranging from 623.97–830.73 mg GAE/100 g, while free phenolics ranged from 106.25–157.35 mg GAE/100 g. After *in vitro* digestion, bioavailable phenolics increased to 801.47 mg GAE/100 g in Tm-T, exceeding the 637.76 mg GAE/100 g value in Tm-C. Antioxidant bioavailability was determined as 49.41 μmol Trolox/g in Tm-T and 41.76 μmol Trolox/g in Tm-C using the ABTS method. In the amino acid profile, the mean of the total amount of essential amino acids was 5.99 g/100 g, with lysine (1.25 g/100 g) and leucine (1.38 g/100 g) being prominent. Amino acids were determined by LC–MS/MS and the essential profile was found to be balanced. Consequently, turmeric-supplemented feeding improves the protein quality of larval powder and the phenolic/antioxidant potential released during digestion, supporting the use of edible insects as carriers of functional components in terms of food innovation.

## 1 Introduction

Nowadays, the positive effects of natural products on health are attracting increasing attention. Plant-derived components have drawn the interest of modern medicine as well as traditional medicine (1). In this context, studies on the potential health benefits of curcumin, an important component of the turmeric plant, have accelerated. Turmeric (*Curcuma longa*), a member of the ginger genus, has roots that are desiccated, pulverized, and extensively employed as a spice. The principal active compound in turmeric (*C. longa*) is curcumin, a polyphenolic substance. Curcumin is a chemical possessing potent anti-inflammatory and antioxidant characteristics. Since ancient times, turmeric and curcumin-containing preparations have been used in traditional Indian medicine and other natural medical practices for the treatment of various health problems. It has wide biological and pharmacological effects (1, 2).

Scientific research indicates that curcumin could have a potential impact in the control of several health disorders. In particular, the anti-inflammatory properties of curcumin have been shown to play a supportive role in the treatment of rheumatoid arthritis, osteoarthritis, and other inflammatory diseases (3). In addition, the antioxidant effects of curcumin may reduce cellular damage by combating free radicals and may reduce the risk of many diseases such as neurodegenerative, cancer, and cardiovascular diseases (4).

Curcumin has been reported to have hepatoprotective, nephroprotective, hypoglycemic, anti-inflammatory, antioxidant, antimicrobial, anticarcinogenic, protective against myocardial infarction, antirheumatic, and thrombosis-suppressing effects (5).

Despite the fact that curcumin has been reported to be safe even at relatively high doses and has such widespread beneficial effects, the greatest limitation restricting its use is its very low bioavailability. This situation may limit the complete absorption of curcumin by the body and its effective utilization. However, scientists have developed various methods to solve this problem. For example, nanoparticle technology has been used to increase the bioavailability of curcumin (6), as well as carrier systems such as microspheres and liposomes (1, 2, 7). These systems may improve bioavailability by increasing the absorption of curcumin in the digestive system. Furthermore, it is evident that natural components, such as piperine, have the capacity to enhance the bioavailability and absorption of curcumin (6). Despite the implementation of various approaches to address this issue, the desired level of curcumin bioavailability has yet to be attained.

Approximately 1,700 insect species are consumed worldwide (8). The swift growth of the population of humans, technological advancement, and the climate problem demand the exploration of alternate sources of nutrition to animal agriculture. Entomophagy, defined as the consumption of insects, is not a new concept for many countries worldwide and refers to popular foods that are traditionally and willingly consumed by local populations (9). The increasing popularity of edible insects in different countries has been driven by the perception that they may serve as a solution in situations of food scarcity. Research indicates that the mealworm (*T. molitor* L.), a significant member of edible insects used especially for human consumption, makes a substantial contribution to human nutrition due to its high nutritional quality (10). Insects are considered a sustainable resource (11), and in recent years, studies on edible insects have increased significantly, focusing not only on their nutritional value and health effects but also on their risks and consumer acceptance (10). The European Commission’s recognition of certain insect species as safe for consumption in European Union member states has paved the way for the marketing of “novel foods” (12). The mealworm (*T. molitor* L.) was the first insect species approved for human consumption as food (13). However, in developed countries, the disgust felt toward insects and the lack of consumer acceptance among the majority of the population have been reported as major barriers to their evaluation as protein sources (14).

Edible insects are regarded as an alternative dietary source. Since 2018, insects have been accepted as novel foods in accordance with Regulation (EU) 2015/2283 (15). The European Union (EU) Commission has approved the marketing of mealworm larvae processed with UV treatment and in powdered form as food and their use in certain products (16). In this context, it is essential to assess this product, intended for sale as human food or as a food additive, in accordance with food safety standards. The breeding of insects with a high protein content for human consumption is a more economical and environmentally-friendly process. Mealworm larvae are a valuable food source for the human organism in more advantageous amounts compared to traditional animal foods such as meat (17). The mealworm (*T. molitor* L.) is a stored-product pest whose larvae are commonly consumed by humans (18). The mealworm is a high-quality product that can be used as a supplement to traditional food sources (19). In the past few decades, mealworm larvae have emerged as an appealing replacement protein resource.

The mealworm (*T. molitor* L.) is an insect species that mostly consumes decomposed plant matter and organic refuse. Due to their structure, although their feeding habits vary, they play an important role as organic matter decomposers in many ecosystems (20). Mealworm larvae contribute to the transformation of environmental materials by breaking down and digesting nutrient sources (21). Especially in the animal feed industry and the field of biotechnology, studies on the use of mealworm larvae as a nutrient source are increasing (22). These studies offer new opportunities to expand the use of mealworm larvae in potential nutritional and medical applications.

It has been reported that mealworms have unique gut microbiomes (23, 24). Mealworm larvae can digest various organic materials and convert them into energy and nutrients (25). In their natural habitats, they feed on various plants and consume the digestible parts of these plants. Turmeric can also be digested by mealworm larvae. The nutrients and components contained in turmeric may potentially be beneficial for the development of mealworm larvae. Nevertheless, research conducted on the digestive system of mealworm larvae with regard to the digestibility of turmeric and the bioavailability of its active components post-digestion is scarce (26). Therefore, more information is needed on how effectively mealworm larvae can digest turmeric and how much of its components can be absorbed.

Our hypothesis is that the processes that will occur while the digestive system of mealworm larvae digests turmeric may increase the bioavailability of turmeric. Our hypothesis is based on the following fundamentals during the digestion process:

Enzymatic degradation, the intestines contain symbiotic microbiota which are integral to the process of digestion (27). The enzymes present in mealworm larvae have been shown to be capable of digesting the components found in turmeric, thereby breaking them down into smaller molecules. This may be carried out by digestive enzymes, as in the human gastrointestinal system. As a result of this breakdown process, the active components of turmeric may become more easily absorbable.

In microbial fermentation, the digestive system of mealworm larvae may interact with the gut microbiota during turmeric digestion (28). This may cause some components of turmeric to undergo microbial fermentation. As a result of fermentation, some components may become more bioavailable or new metabolites may be formed. The effect of carrier proteins on the digestive system of mealworm larvae may cause the active components found in turmeric to interact with carrier proteins. These carrier proteins may enable easier absorption of turmeric by the digestive system or transport it to targeted tissues. In this way, the bioavailability of turmeric may increase. The objective of the study is to determine the fundamental chemical, microbiological, and antioxidant characteristics of powdery mealworm (*T. molitor* L.) larvae as a promising nutritional source for humans.

## 2 Materials and methods

### 2.1 Rearing and feeding

A total of 100 insects (*T. molitor*) were obtained from a commercial live animal supplier. The insects were divided into two groups of 50 individuals each and placed into separate containers.

The insects were maintained at room temperature (25 °C) in containers measuring 40 × 30 × 25 cm. A homogeneous substrate consisting of 25% wheat flour, 25% corn flour, and 50% wheat bran was prepared. To meet their moisture requirements, fresh potato (20 g per colony) was added. The fresh potato slices were replaced daily (29).

Upon the onset of reproduction (10–12 days), the larvae were transferred to a larval rearing container. When the separated larvae reached a length of 25–30 mm (17) (approximately 2 months, 500 g, 5,000 pieces live mealworms per box), they were divided into control and experimental groups, each consisting of eight colonies.

### 2.2 Experimental groups

*Control Group:* Larvae were fed fresh potato supplementation.
*Experimental Group:* Larvae were fed turmeric (*ad libitum*), (20 g daily).

### 2.3 Sample preparation

Following the two-week feeding period, mealworm larvae from both the control and experimental groups were dried in a dry-air oven at 50 °C. The dried mealworm larvae were subsequently pulverized into a powder. The parameters for analysis in the mealworm larvae powders are provided above.

### 2.4 Chemical composition analysis

The moisture and ash content in the research materials were determined using Methods 935.29 and 942.05, respectively (30). Nitrogen analysis was performed according to the AOAC 950.48 protocol to determine protein content as a percentage of dry matter. These data, obtained using the Kjeldahl technique, were converted to protein values using a general coefficient of 6.25 (31). The oil content of the Mealworm samples and turmeric was determined using the Soxhlet system according to AOAC Method No 948.22 (31). The present study utilized the following equations to derive the carbohydrate (32) and energy (33) values of the samples:


Energy(kcal)=4×[Protein(g)+Carbohydrate(g)]+9×[Fat(g)]

%Carbohydrate=100−(Moisture%+Ash%+Protein%+Fat%)

### 2.5 Total phenolic compound extraction

The total phenolic content (TPC) in mealworm and turmeric samples was assessed by extracting both free (soluble) and bound (insoluble) phenolic compounds. The technique described by Vitali et al. (34) was adapted for the extraction of free phenolic chemicals. In summary, 2.0 g of the sample was measured, and 20 mL of a concentrated HCl/methanol/water solution (1:80:10, v/v/v) was included. The mixture was then agitated for 2 h at 20 °C utilizing a multi-tube shaker (Heidolph Multi Reax, Germany). Upon completion of the experimental period, the samples were centrifuged at 3500 rpm for 10 min at 4 °C using a centrifuge (Eppendorf 5,430 R, United States). The isolated supernatants were preserved at −20 °C until analysis. The residue from the extraction of free phenolic compounds was subsequently used to isolate bound phenolic compounds. Twenty milliliters of a 10:1 methanol/sulfuric acid mixture were added to the acquired residue, which was then incubated in a shaking water bath at 85 °C for 20 h. Post-incubation, the study materials were equilibrated to room temperature and centrifuged (4 °C, 3500 rpm, 10 min; Eppendorf 5,430 R, United States), with the resulting supernatants preserved at −20 °C until analysis.

### 2.6 Determination of total phenolic content

The phenolic content, including free and bound fractions, of extracts from mealworm and turmeric samples was determined using the Folin–Ciocalteu colorimetric method, following Naczk and Shahidi (35) and Vitali et al. (34). These findings were presented as gallic acid equivalents (mg GAE/100 g). The Lowry C solution was prepared by combining Lowry A (2% Na₂CO₃ dissolved in 0.1 mol/L NaOH) and Lowry B (0.5% CuSO₄ dissolved in 1% NaKC₄H₄O₆) solutions in a 50:1 (v/v) ratio. The Folin–Ciocalteu reagent was diluted with distilled water at a 1:3 (v/v) ratio, and gallic acid standard solutions ranging from 5 to 50 mg/L were used to establish the calibration curve. Suitable sample dilutions were established during the study based on the colorimetric response. A curve of calibration was created utilizing gallic acid standard solutions within a concentration range of 5–50 mg/L, and the absorbance of mealworm and turmeric extracts was assessed at 750 nm, using a UV–Vis spectrophotometer (Shimadzu UV–Vis 1800, Kyoto, Japan).

### 2.7 Total antioxidant activity

The antioxidant activity of the mealworm and turmeric samples was evaluated using three assays: ABTS^•+^, CUPRAC, and DPPH^•^. The method reported by Apak et al. (36) was applied to analyze the samples using the ABTS^•+^ method. The ABTS^•+^ standard solution (7 mM aqueous solution) used in the analysis was mixed with 2.45 mM K_2_S_2_O_8_ and incubated in the dark for 12–16 h. The resulting ABTS^•+^ working solution was diluted 1:10 with 96% ethanol. An aliquot (x mL) of each extract (free and bound) was mixed with (4-x) mL of ethanol and 1 mL of the ABTS^•+^ solution. The mixture was vortexed thoroughly and incubated in the dark for 6 min. Subsequently, the absorbance was measured at 734 nm using a spectrophotometer (Shimadzu UV–Vis 1800, Kyoto, Japan) (A_sample_). The same procedures were performed without adding the extract as a control sample, and the absorbance value was read (A_control_). The % inhibition values were calculated as follows, based on the measurements:


$$\text{Inhibition}\%=[\frac{\text{Acontrol}-\text{Asample}}{\text{Acontrol}}]\times 100$$

The calibration curve was constructed using Trolox standard solutions in the concentration range of 0.00625–0.05000 mg, and the results were expressed as μmol Trolox equivalents per gram of dry sample (μmol TE/g).

Total antioxidant activity of the research materials was determined using the CUPRAC assay, as described by Apak et al. (37). The three main stock solutions required for the analysis were prepared as follows: At the beginning, 0.4262 g of CuCl_2_ was dissolved in 100 mL of distilled water to yield a concentration of 1.0 × 10^−2^ M copper(II) chloride. Following that, 0.0390 g of neocuproine (C₁₄H₁₂N₂) was dissolved in 96% ethanol and diluted to a final volume of 25 mL, resulting in an amount of 7.5 × 10^−3^ M. Furthermore, 19.27 g of ammonium acetate (NH₄Ac) was dissolved in distilled water and diluted to a final volume of 250 mL to create a 1 M solution of buffer.

One mL of each solution prepared for spectrophotometric measurements was transferred to Falcon tubes. Blind (control) samples, containing no extract, were also prepared using the same procedure. Following incubation at room temperature for 30 min, the absorbance of the samples was measured at 450 nm using a UV–Vis spectrophotometer (Shimadzu UV-1800, Japan). The CUPRAC antioxidant activity of the extracts was calculated as μmol Trolox equivalents per gram of dry sample (μmol TE/g) using the calibration equation.

The total antioxidant activity of mealworm and turmeric samples was determined using the 2,2-diphenyl-1-picrylhydrazyl radical scavenging method, as previously detailed by Brand-Williams et al. (38). To prepare a DPPH^•^ stock solution at a concentration of 1 × 10^−3^ M, 0.039 g of DPPH^•^ was dissolved in methanol, and the volume was adjusted to 100 mL. Subsequently, 6.0 mL of this stock solution was diluted to 100 mL with methanol to obtain a working solution with a final concentration of 6 × 10^−5^ M for use in the experiments.

Trolox standards were prepared in volumes of 10, 25, 50, 75, and 100 μL, to which DPPH• solution was added, resulting in a total volume of 4 mL. To enable the reaction to reach a steady state and determine the maximum absorbance, the mixtures were incubated in darkness and observed at 5-min intervals for 5 to 30 min.

After incubation, the density of light of the sample (A_sample) and control (A_control) solutions at 515 nm was measured using a Shimadzu UV–Vis 1800 instrument from Kyoto, Japan. The percentage inhibition (%) indicative of radical-scavenging activity was calculated from the absorbance data.


$$\text{Inhibition}\%=[\text{Acontrol}-\frac{\text{Asample}}{\text{Acontrol}}]\times 100$$

The DPPH^•^ solution was added to the sample, as determined by preliminary tests, and left in the dark for the duration of the experiment, as previously determined by preliminary tests. Following this, the absorbancy values were measured (A_sample_). The antioxidant activities of the samples were calculated as μmol Trolox/g sample using the calibration graph.

### 2.8 Bioaccessibility of total phenolic content and antioxidants

The extraction efficiency of phenolic compounds from food matrices is influenced by several factors, including the composition of the food matrix, the extraction method, particle size, and the presence of interfering substances. Therefore, the extraction method, solvent, and techniques suitable for the samples to be analyzed were determined based on preliminary tests. The bioaccessibility of TPC and antioxidants was determined with slight modifications according to the methods described by Vitali et al. (34) and Naczk and Shahidi (35). For this purpose, an *in vitro* enzymatic digestion procedure simulating gastrointestinal conditions, including gastric and intestinal phases, was carried out under controlled laboratory conditions.

The gastrointestinal digestion simulation began with the gastric phase, in which 2 g of the sample was mixed with 10 mL of distilled water and 0.5 mL of pepsin solution. The pH of the mixture was adjusted to 2.0 using 5 mol/L HCl, and the samples were incubated at 37 °C for 1 h in a shaking water bath. Then, to proceed to the intestinal phase, the pH was equilibrated to 7.2 with 1 M NaHCO_3_; the process continued with the addition of 2.5 mL of bile/pancreatin and 2.5 mL of NaCl/KCl solutions. This final mixture was incubated again at 37 °C for 2.5 h. At the end of the digestion period, the supernatants were separated by centrifugation (Eppendorf 5,430 R, United States; 4,000 rpm, 10 min). The TPC, antioxidant activity, and bioavailability rates of the relevant components were analyzed from these extracts.

The bioaccessibility values of the extracts were calculated using the corresponding calibration equations. The total phenolic content (TPC) was quantified as milligrams of gallic acid equivalents per 100 grams of sample, utilizing the calibration equation (y = ax ± b; R^2^). In contrast, antioxidant activities assessed through the ABTS•^+^, CUPRAC, and DPPH• tests were reported as micromoles of Trolox equivalents per gram of sample (μmol TE/g).

### 2.9 Determination of free amino acid composition (LC–MS)

The amino acid content was examined following the methodology of Bilgin (39). This study utilized an LC–MS/MS (Liquid Chromatography–Tandem Mass Spectrometry, Agilent 6,460, Agilent Technologies, Waldbronn, Germany) equipment for AA analyses. The samples underwent acid hydrolysis (4 mL HCl solution, 110 °C for 24 h), and the supernatant was then obtained through centrifugation. Material preparation and evaluation were performed using an Agilent Infinity 1,260 HPLC system coupled to a 6,460 triple quadrupole mass spectrometer, with Jasem LC–MS/MS amino acid kits. Nineteen amino acids were analyzed, including seven essential (methionine, isoleucine, leucine, threonine, phenylalanine, valine, and lysine), two semi-essential, and 10 non-essential, utilizing peak area ratios in relation to the corresponding internal standards. The parameters for the mass detector included a gas temperature of 150 °C, a gas flow rate of 10 L/min, a nebulizer pressure of 40 psi, and a capillary voltage of +2000 V. Each sample underwent duplicate analysis. The measurements are expressed in grams per 100 grams.

### 2.10 Determination of fatty acid composition

The fatty acids that were extracted from turmeric and *T. molitor* larvae were determined to be Palmitic (C16:0), Palmitoleic (C16:1), Stearic (C18:0), Oleic (C18:1), Linoleic (C18:2), Linolenic (C18:3), Arachidic (C20:0), Behenic (C22:0), and Lignoseric (C24:0). After carrying out oil derivatization operations, the samples were subjected to oil extraction using the Soxhlet technique (40). The samples were then examined using a Shimadzu GC-17A gas chromatograph with a flame ionization detector. A 10 mL screw-cap test tube was used for sample pretreatment. 0.1 g of the test sample was measured into the container, 2 mL of isooctane was added, and the mixture was stirred. After that, 0.1 mL of a 2 mol/L methanolic potassium hydroxide solution was added, the PTFE tube cover was fastened, and the mixture was vigorously mixed for 1 min. As the glycol separated, the solution went from clear to rather cloudy. Following a 2-min standing time, 2 mL of NaCl solution was added and mixed with a vortex. Roughly 1 g of sodium hydrogen sulfate was then added and mixed with the vortex. The top phase was moved into a gas chromatography vial and examined with a gas chromatograph equipped with an Agilent 6,890 N gas chromatograph, an ECD/FID detector, and a Split/Splitless control. You may find the details of the GC-FID FAME analysis in Supplementary Table S1. Standard fatty acid retention times were compared to these to determine the fatty acid composition in compliance with ISO 2015 (Method: TS EN ISO12966-4). The fatty acid composition was determined by Sigma Aldrich of St. Louis, MO, United States. A gas chromatography system from Agilent, United States, model 6,890 N, which includes a flame ionization detector and an HP-88 capillary column (100 m × 0.25 mm × 0.20 μm), was used to analyze fatty acid methyl esters (FAME) in the C4-C24:1 range.

This gas chromatography analysis made use of injector and detector temperatures of 250 °C and 300 °C, respectively. The DB-WAX capillary column (30 m × 0.25 mm I. D. and 0.25 μm film thickness) was used in inverse analysis with split injection mode (1:25) to quantify fatty acid methyl esters. Detector for Ionization of Flames The FID was operated at a flow rate of 45 mL/min of hydrogen, 450 mL/min of dry air, and 45 mL/min of nitrogen as the carrier gas. The temperature in the oven was raised to 220 °C in steps of 20 °C per minute, after which there was a 45-min hold at 120 °C. Helium was used as the carrier gas, and a flow rate of 1 mL/min was used. The overall analysis time was 53 min and the injection amount was 1 μL.

### 2.11 Mineral determination (ICP–MS)

To minimize matrix-induced interference in multi-element quantification, samples were subjected to microwave-assisted acid digestion prior to instrumental analysis. A precisely weighed portion of the homogenized sample was placed in Teflon digestion vessels, and high-purity concentrated nitric acid and hydrogen peroxide were added at a ratio of HNO₃:H₂O₂ = 9:3 (v/v). Digestion was performed using a Milestone SK10 microwave system at maximum power, 220 °C for 20 min, and a maximum pressure of 45 bar. After cooling to room temperature, the digestion products were quantitatively transferred to pre-cleaned 50.0 mL polypropylene tubes and volume-completed (50 mL) with ultrapure water; extrapure water was used as the sole diluent throughout sample preparation. Element concentrations were determined using ICP-MS (Agilent 7,700X, United States). Quantification was performed via external calibration using a multi-element standard solution (Agilent 8,500–6,940 2A; 27-element mixture) to generate calibration curves. Method performance (accuracy, sensitivity, recovery, and detection limit) was validated through QA/QC procedures involving a triple analysis of a certified reference material (NIST 2702). Results were expressed in mg/kg (ppm equivalent) according to the reporting format used in the mineral table.

### 2.12 Statistical analysis

Data obtained from the mealworm samples were analyzed by analysis of variance (ANOVA) using JMP 11 software (JMP Statistical Discovery, SAS Institute Inc., Cary, NC, United States). Differences among mean values were evaluated using the Tukey HSD test at a significance level of *p* < 0.05. Relationships between variables were examined using Pearson correlation analysis; *p*-values in multiple tests were corrected using the Benjamini–Hochberg (BH) method. To reveal multivariate patterns, all variables were analyzed using PCA after z-score standardization (centering and scaling); hierarchical clustering analysis (HCA) was also performed using Euclidean distance and Ward.D2 coupling method, and the results were visualized as a clustered heat map.

## 3 Findings and discussion

### 3.1 Chemical analysis

The results of the chemical composition analysis (moisture, crude ash, crude protein, crude fat, carbohydrate, and energy value) of mealworm (*T. molitor* L.) larvae fed with turmeric (Tm-T), control larvae (Tm-C), and fresh turmeric (T) samples (Supplementary Figure S1) are presented in Table 1. According to the results, the moisture content of the Tm-C samples (64.38%) was found to be significantly higher (*p* < 0.05) than that of the Tm-T samples (61.77%) while the ash content of the Tm-C, Tm-T, and T samples was determined to be 1.40, 1.65, and 1.70%, respectively.

**Table 1:** Proximate composition and energy values of *T. molitor* larvae and fresh turmeric samples (mean ± SD).

Sample	Moisture^1^ (%)	Ash^1^^*^ (%)	Protein^1^^,^^2^^*^ (%)	Fat^*^ (%)	Carbohydrate (%)	Energy (kcal)
Tm-C	64.38 ± 0.08^a^	1.40 ± 0.03^a^	21.61 ± 0.03^b^	9.04 ± 0.82^a^	3.56 ± 0.84^b^	182.06 ± 3.86^a^
Tm-T	61.77 ± 0.11^b^	1.65 ± 0.09^a^	24.04 ± 0.09^a^	6.84 ± 0.14^b^	5.07 ± 0.03^a^	180.51 ± 1.48^b^
T	74.99 ± 2.66	1.70 ± 0.08	1.08 ± 0.01	0.25 ± 0.99	23.92 ± 2.91	102.27 ± 1.48

*Estimated dry weight basis.

Tm-C, Control; Tm-T, Mealworm fed with turmeric; T, Turmeric.

1 Values are averages of three replicates and are given with standard deviations.

2 Nx6.25.

Statistics were performed on mealworm samples. When comparing the means of different letters in a given row (a–b), a significant difference between the groups was indicated by the Tukey HSD test (*p* < 0.05).

The significantly higher protein content observed in the Tm-T sample (24.04%) compared with the Tm-C sample (21.61%) (*p* < 0.05) suggests that turmeric supplementation may have contributed to enhanced protein accumulation in the mealworm samples. Similar protein contents for *T. molitor* larvae have been reported in previous studies, generally ranging between 18 and 25% on a fresh weight basis, depending on diet composition and rearing conditions (41). The increase in protein content observed in the Tm-T samples may be attributed to the bioactive compounds present in turmeric.

In the present study, the carbohydrate content of the Tm-C and Tm-T samples was determined to be 3.56 and 5.07%, respectively. Although insects generally contain low levels of carbohydrates, variations may occur depending on dietary fiber and secondary metabolites present in the feed. The higher carbohydrate content observed in Tm-T samples may be related to the carbohydrate-rich structure of turmeric.

On the other hand, when the fat content of the samples was examined, it was found that the fat content of Tm-T (6.84%) was statistically significantly lower (*p* < 0.05) than that of Tm-C samples (9.04%). Previous studies have shown that dietary composition strongly affects fat accumulation in *T. molitor* larvae, with reported fat contents ranging from 5 to 15% (42, 43). The reduction in fat content in Tm-T may be associated with turmeric’s potential hypolipidemic effects, which have been reported to modulate lipid metabolism in various organisms.

When these results obtained in the presented study are evaluated in general, they show that turmeric supplementation significantly altered the composition of *T. molitor* larvae by reducing fat content while increasing protein and carbohydrate content. These findings suggest that turmeric can be used as a functional feed ingredient to increase the nutritional value of edible insects.

### 3.2 Total phenolic content and antioxidant activity of free, bound, total fractions and *in-vitro* bioaccessible phenolics

**Figure 1:**
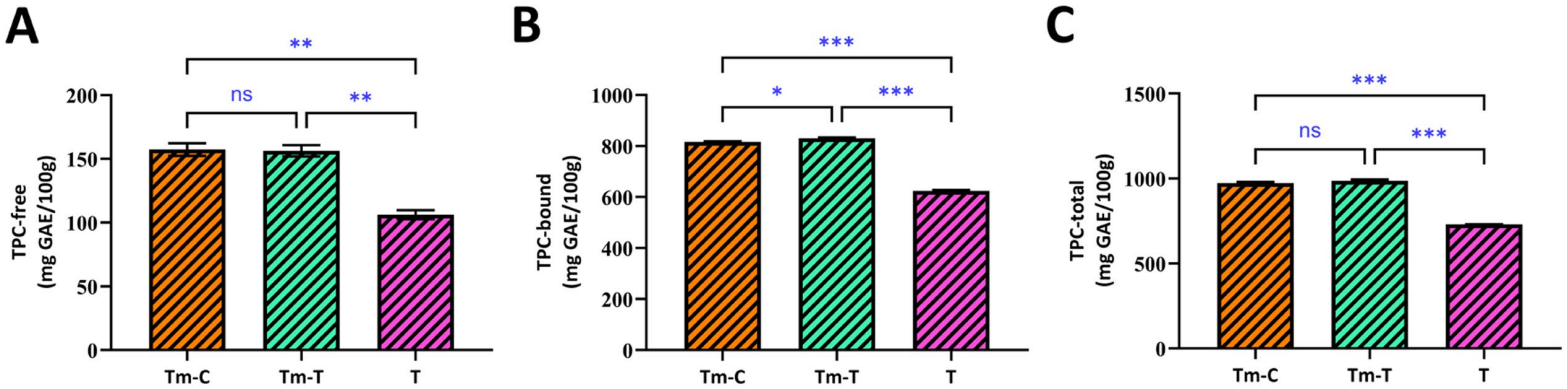
Free **(A)**, bound **(B)**, and total **(C)** total phenolic content (TPC; mg GAE/100 g) in *T. molitor lar*vae and turmeric samples (T, turmeric root; Tm-C, mealworm control; Tm-T, mealworm fed with turmeric). The data are expressed as the mean ± standard deviation (*n* = 3). Brackets indicate pairwise comparisons; ns indicates no significant difference (*p* > 0.05), whereas *, **, and *** indicate *p* < 0.05, *p* < 0.01, and *p* < 0.001, respectively.

The amounts of free (extractable), bound (hydrolysable), and total phenolic compounds in the research materials are given in Figure 1. The results were calculated according to the calibration graph given in Supplementary Figure S2 using the equation y = 0.0461x + 0.0404, R^2^ = 0.9472 in gallic acid equivalent. Accordingly, the amount of bound phenolic compounds in the samples, ranging from 623.97 to 830.73 mg GAE/100 g, was found to be higher than the amount of free phenolic compounds (106.25–157.35 mg GAE/100 g). Previous studies have reported that in many food matrices, including cereals, insects, and plant-derived feeds, phenolic compounds are predominantly present in the bound form, covalently linked to cell wall components such as hemicellulose, proteins, and cellulose (44, 45).

Within the scope of the data we obtained, the *in-vitro* bioaccessible phenolic content of the TM-C samples in our study was 637.76 mg GAE/100 g, while this value was 801.47 mg GAE/100 g in the TM-T samples, which were statistically significantly different (*p* < 0.05). The increase in Tm-T can be attributed to turmeric’s high phenolic content and the release of bound phenolics during digestion. In a study by Nisar et al. (46), flour moth samples were extracted using various solvents, including 60 and 80% ethanol, 60 and 80% methanol, as well as water. The total phenolic content was reported as 78.76, 745.76, 53.87, 682.43, and 496.76 mg GAE/100 g for the respective extraction solvents. The wide range determined by Nisar et al. (46) clearly demonstrates that the solvent used in extraction has a strong effect. However, the higher phenolic values obtained in the present study can be attributed to the inclusion of bound phenolic fractions and to *in vitro* digestion, which releases phenolics that are not accessible by traditional solvent extraction. Overall, the results indicate that bound phenolic compounds represent the dominant phenolic fraction in *T. molitor* larvae, and dietary supplementation with turmeric significantly enhances the bioaccessible phenolic content. This suggests that the nutritional and functional value of edible insects can be modulated through phenolic-rich feed ingredients.

**Figure 2:**
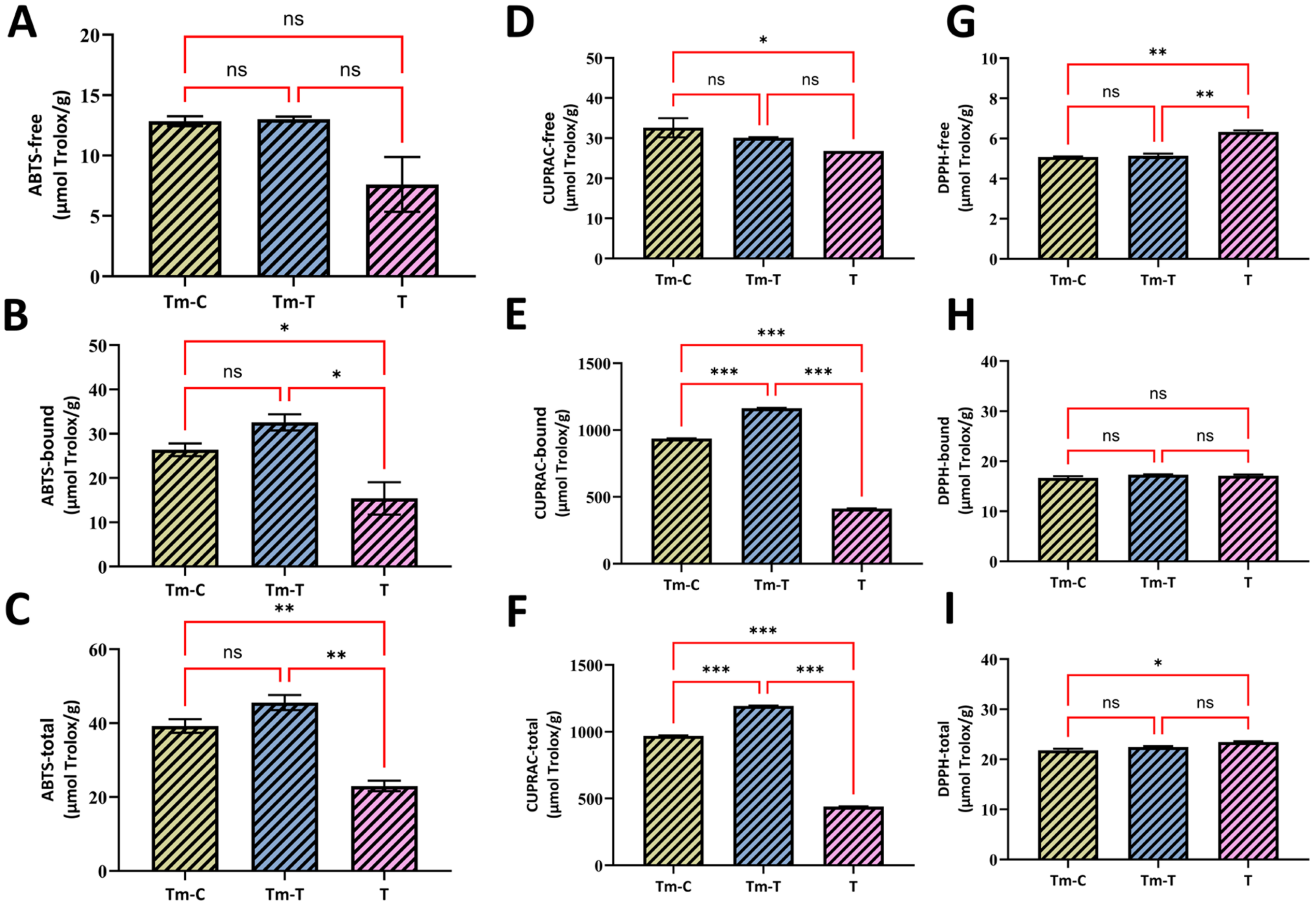
Antioxidant activity (μmol Trolox/g) of free, bound, and total fractions measured by ABTS **(A–C)**, CUPRAC **(D–F)**, and DPPH **(G–I)** assays in the samples T (turmeric root), Tm-C (mealworm control), and Tm-T (mealworm fed with turmeric). The data are expressed as the mean ± standard deviation (*n* = 3). Brackets indicate pairwise comparisons; ns indicates no significant difference (*p* > 0.05), whereas *, **, and *** indicate *p* < 0.05, *p* < 0.01, and *p* < 0.001, respectively.

Antioxidant activity is influenced by multiple factors; therefore, various methods have been developed to evaluate it. The antioxidant activities of the research material samples were analyzed using the ABTS^•+^, CUPRAC, and DPPH^•^ methods, and the results are presented in Figure 2. The bound antioxidant activity values of turmeric samples used to feed mealworm samples were found to be higher than their free antioxidant activities in all methods (ABTS^•+^, CUPRAC, and DPPH^•^). Accordingly, the highest antioxidant activity in turmeric samples was detected in bound phenolics (413.04 μmol trolox/g) using the CUPRAC method, while the lowest antioxidant value was detected in free phenolics (6.33 μmol trolox/g) using the DPPH^•^ method (Figure 2). When these results are evaluated, it is revealed that bound phenolic compounds play a dominant role in turmeric’s antioxidant activity and that the CUPRAC method is effective for detecting this activity.

The antioxidant activities of free and bound phenolics extracted from research materials were analyzed using the ABTS^•+^ method. Based on the calibration curves drawn (y = 1054.3x + 19.251, R^2^ = 0.9824), the ABTS^•+^ antioxidant activity values of the extracts were calculated as μmol trolox/g sample (Supplementary Figure S3). The difference between the two samples was not statistically significant (*p* ˃ 0.05), even though the ABTS• + antioxidant activity of free phenolics in the Tm-C sample (12.84 μmol trolox/g) was lower than in the Tm-T samples (13.01 μmol trolox/g). However, the turmeric samples had an antioxidant activity of 7.62 μmol trolox/g. The bound phenolics’ antioxidant capabilities were observed to differ significantly, with the mealworm samples fed turmeric showing the greatest antioxidant activity value (32.55 μmol trolox/g) (Figure 2). The observation of higher ABTS^•+^ antioxidant activity in the bound phenolic fraction of turmeric-fed larvae is consistent with the high bound phenolic content in these samples. Overall, these findings indicate that turmeric supplementation particularly increases the bound phenolic fraction and significantly increases ABTS^•+^ antioxidant activity, while free phenolics are relatively unaffected.

The antioxidant activity results for the research materials are presented in Figure 2. The CUPRAC method results were calculated using the equation y = 15.377x + 0.0163, with R^2^ = 0.9974, based on the calibration graph provided in Supplementary Figure S4. Although the free phenolic fractions of the Tm-T samples had lower CUPRAC antioxidant activity (30.11 μmol trolox/g) than the Tm-C samples (32.60 μmol trolox/g), the antioxidant activities of the bound phenolics of the TM-T samples (1162.81 μmol trolox/g) were found to be higher than those of the Tm-C samples (936.54 μmol trolox/g) (Figure 2). A statistically significant difference (*p* < 0.05) was observed between these values.

Results for the DPPH^•^ method were calculated using the equation y = 3537.4x + 2.0174, R^2^ = 0.9972, equivalent to trolox, according to the calibration graph provided in Supplementary Figure S5. No statistically significant differences were found among the samples (*p* > 0.05), and the antioxidant activity of free phenolics in the DPPH• technique was found to be 5.07 μmol trolox/g in the Tm-C samples and 5.14 μmol trolox/g in the TM-T samples (Figure 2). Similarly, the DPPH• technique revealed that the bound phenolics in the TM-T samples had a higher antioxidant activity (17.32 μmol trolox/g) compared to the control samples (16.69 μmol trolox/g) (Figure 2), but this difference was not statistically significant (*p* < 0.05). Previous studies have reported that the DPPH^•^ method is less sensitive to hydrophilic antioxidants and complex phenolic structures, and that this generally leads to lower antioxidant activity values and limited differentiation between samples (47, 48).

### 3.3 *In-vitro* bioaccessibility

In the present study, the *in vitro* bioaccessibility of the research material samples was analyzed using the ABTS^•+^, CUPRAC, and DPPH^•^ methods, and the results are presented in Table 2. In the ABTS^•+^ assay, the antioxidant bioaccessibility value of the Tm-C samples was determined as 41.76 μmol trolox/g, while this value was 49.41 μmol trolox/g in the Tm-T samples. A statistically significant difference was observed among the samples (*p* < 0.05). In the CUPRAC assay, the antioxidant bioaccessibility value of the Tm-C samples was 50.51 μmol trolox/g, whereas this value was 44.05 μmol trolox/g in the Tm-T samples. For the DPPH^•^ assay, the antioxidant value of the bioaccessibility for the Tm-C samples was of 7.67 μmol trolox/g and in case Tm-T it reached up to 5.98 μmol trolox/g.

**Table 2:** *In-vitro* bioaccessibility values.

Sample	ABTS (μmol trolox/g)	CUPRAC (μmol trolox/g)	DPPH (μmol trolox/g)
Tm-C	41.76 ± 1.54ᵇ	50.51 ± 6.34ᵃ	7.67 ± 0.96ᵃ
Tm-T	49.41 ± 0.69ᵃ	44.05 ± 5.21ᵇ	5.98 ± 1.54ᵇ
T	38.31 ± 4.05	2.79 ± 0.16	0.79 ± 0.06

Tm-C, Mealworm control; Tm-T, Mealworm fed with turmeric; T, Turmeric root; The data are expressed as the mean ± standard deviation (*n* = 3). Statistics were performed on mealworm samples. When comparing the means of different letters in a given row (a–b), a significant difference between the groups was indicated by the Tukey HSD test (*p* < 0.05).

Phenolic compounds can be converted from bound forms into free form by alkaline, acidic, or fermentation practices (49). While the free phenolic content of mealworms fed with turmeric (156.30 mg GAE/100 g) was slightly lower compared to the control one (157.35 mg GAE/100 g), higher bound phenolic content in Tm-T samples (830.73 mg GAE/100 g) significantly increased the total amount of phenolics, which was statistically significant (*p* < 0.05). The TP compositions of the samples also varied significantly (*p* <  0.05).

There are no studies in the literature on mealworms fed turmeric directly. It has been reported that insects possess diverse gut microbiomes and extracellular enzyme systems capable of degrading complex plant matrices, thereby releasing and converting bound phenolic compounds (50). The increase in total phenolic compound content in the Tm-T samples can be attributed to the breakdown of turmeric’s phenolic compounds and the release of bound phenolics by the mealworms’ extracellular enzyme systems and gut microbiota.

Digestibility and bioaccessibility are key parameters that determine the nutritional and functional value of edible insects. While digestibility refers to the breakdown of nutrients, bioaccessibility reflects the fraction that becomes available for absorption and physiological function. As emphasized by Imathiu (14), nutrients that are poorly bioavailable do not confer expected health benefits, regardless of their abundance in the food matrix. In this context, the increase in *in-vitro* bioaccessible phenolic content in Tm-T samples suggests a potential enhancement in the functional quality of insect-based foods.

Turmeric is widely recognized for its high curcumin content; however, curcumin exhibits notoriously low oral bioavailability due to poor solubility, rapid metabolism, and extensive fecal excretion (51, 52). In the present study, we hypothesize that processes in the digestive system of mealworm larvae during turmeric digestion may increase turmeric bioavailability. The results obtained in the present study provide evidence supporting this hypothesis. In our study, while the *in-vitro* bioavailability phenol content of the control samples was 637.76 mg GAE/100 g, this value was 801.47 mg GAE/100 g in the TM-T samples.

The control samples had an antioxidant bioaccessibility of 41.76 μmol trolox/g when tested using the ABTS• + technique, whereas the TM-T samples had 49.41 μmol trolox/g. According to Table 2, a difference that was statistically significant (*p* < 0.05) was found. According to comprehensive research conducted by Sanidad et al. (52), curcumin has low oral bioavailability and a suboptimal pharmacokinetic and pharmacodynamic profile. Similarly, as suggested by Metzler et al. (51), numerous animal studies have reported that 90% of orally administered curcumin is excreted via feces.

### 3.4 Amino acid composition and protein quality

The amino acid (AA) contents of the mealworm control group (Tm-C), mealworm (*T. molitor* L.) larvae fed with turmeric (Tm-T), and root turmeric (T) samples are presented in Table 3. All samples contained varying amounts of the amino acids histidine, phenylalanine, arginine, alanine, proline, aspartic acid, ornithine, leucine, glutamic acid, tyrosine, isoleucine, lysine, valine, methionine, threonine, and serine. Cystine and taurine were found at low levels and were not detected in some groups (*p* < 0.05).

**Table 3:** Amino acid composition (g/100 g) and one-way ANOVA results for the three samples (Tm-C, Tm-T, T).

No	Amino acid (g/100 g)	Tm-C (g/100 g)	Tm-T (g/100 g)	T (g/100 g)
1	Alanine	1.76 ± 0.12^a^	1.78 ± 0.11^a^	1.82 ± 0.11^b^
2	Arginine	0.98 ± 0.10^b^	1.00 ± 0.10^a^	1.01 ± 0.10^c^
3	Aspartic Acid	1.31 ± 0.16^b^	1.67 ± 0.16^a^	1.69 ± 0.15^c^
4	Cystine	0.10 ± 0.00^b^	0.11 ± 0.00^a^	0.11 ± 0.00^c^
5	Glutamic Acid	2.66 ± 0.01^b^	2.82 ± 0.01^a^	2.83 ± 0.01^c^
6	Glycine	1.16 ± 0.19^b^	1.21 ± 0.19^a^	1.25 ± 0.19^c^
7	Histidine	0.69 ± 0.04^a^	0.69 ± 0.04^a^	0.68 ± 0.04^c^
8	Isoleucine	0.50 ± 0.02^a^	0.47 ± 0.02^b^	0.47 ± 0.02^c^
9	Leucine	1.44 ± 0.09^a^	1.38 ± 0.07^b^	1.38 ± 0.07^c^
10	Lysine	1.24 ± 0.10^a^	1.26 ± 0.10^a^	1.25 ± 0.10^b^
11	Methionine	0.27 ± 0.02^a^	0.27 ± 0.02^a^	0.26 ± 0.02^b^
12	Ornitine	0.35 ± 0.29^a^	0.31 ± 0.29^b^	0.30 ± 0.29^c^
13	Phenylalanine	0.80 ± 0.07^a^	0.77 ± 0.07^a^	0.80 ± 0.07^b^
14	Proline	1.50 ± 0.03^a^	1.49 ± 0.03^a^	1.49 ± 0.03^b^
15	Serine	0.95 ± 0.05^b^	1.10 ± 0.05^a^	1.06 ± 0.05^c^
16	Threonine	0.84 ± 0.06^b^	0.90 ± 0.06^a^	0.89 ± 0.06^c^
17	Tyrosine	1.25 ± 0.12^b^	1.43 ± 0.12^a^	1.42 ± 0.12^c^
18	Valine	0.96 ± 0.08^a^	0.95 ± 0.08^a^	0.92 ± 0.08^b^
19	Taurine	0.03 ± 0.03^a^	0.03 ± 0.03^a^	0.03 ± 0.03^a^

Tm-C, Mealworm control; Tm-T, Mealworm fed with turmeric; T, Turmeric root; n.d., not determined. The data are expressed as the mean ± standard deviation (*n* = 3). When comparing the means of different letters in a given row (a–c), a significant difference between the groups was indicated by the Tukey HSD test (*p* < 0.05). ANOVA, one-way analysis of variance; SD, standard deviation.

In terms of total amino acid level, the highest amino acid results were detected in Glutamic acid samples Tm-T (2.83 g/100 g) and Tm-C (2.66 g/100 g). Glutamic acid, alanine, proline, leucine, and glycine are the amino acids found at the highest levels in mealworm larvae (*T. molitor L.*) samples (*p* < 0.05). This amino acid profile is thought to affect both nutritional quality and functional properties.

The analysis of mealworm larvae samples that were given turmeric showed that the average total essential amino acid content, denoted as *Σ*-EAA (The total amount of essential amino acids- lysine, isoleucine, leucine, phenylalanine, methionine, valine, and threonine), was 5.99 g/100 g. Notably, the levels of lysine (1.25 g/100 g) and leucine (1.38 g/100 g) were higher than the other essential amino acids, which were distributed evenly. In terms of protein quality, mealworms that are fed turmeric seem to provide a high nutritional value.

The larvae fed on turmeric T-fed mealworm (Tm-T) contained significantly higher levels of serine, glutamic acid, aspartic acid, tyrosine, and threonine amino acids compared to those in Tm-C and root turmeric (T). This difference can be explained on the basis of biologically active components including curcumin and phenolic compounds in turmeric, which help in increasing the protein metabolism, thereby reducing degradation of amino acid. These results correspond to those compiled from the literature, while Meyer-Rochow et al. (53) reporting that amino acid levels and bioavailability in edible insects are dependent upon dietary substrate, time of harvest and processing techniques implemented.

In addition to their role in building umami flavor, amino acids are vital for proper nutrition. Kurihara (54) reports that glutamic acid is one of the most common amino acids in foods, is the primary determinant of umami flavor, and remains highly stable against heat; furthermore, free glutamate levels increase during processes such as fermentation. Here, it is believed that mealworm metabolism was supported by the phenolic chemicals and curcumin found in turmeric, leading to an increase in glutamate production and tissue buildup. The significantly high levels of glutamic acid observed in turmeric fed samples from our study suggests a possible benefit with regard to umami flavor contribution and could be used as leads for product development studies. Jajić et al. (55) determined the amino acid profile of larvae on a dry matter basis, focusing on glutamic acid (11.54 g/100 g protein), aspartic acid (7.71 g/100 g protein), alanine (8.11 g/100 g protein), isoleucine (7.38 g/100 g protein), and tyrosine (6.92 g/100 g protein). With appropriate levels of key amino acids like methionine (3.16 g/100 g protein), leucine (5.31 g/100 g protein), and lysine (4.79 g/100 g protein), mealworms could be an alternate protein source for animal feed and human nutrition.

Mealworm larvae (*T. molitor* L.) samples showed an amino acid profile that is very close to the adult requirements indicated by FAO/WHO/UNU (56). Our results showed that the reference patterns were borne out by the values of leucine (1.38–1.44 g/100 g protein), isoleucine (0.47–0.50 g/100 g protein), threonine (0.84–0.90 g/100 g protein), and lysine (1.24–1.26 g/100 g protein). The sum of phenylalanine + tyrosine (~2.0–2.2 g/100 g protein) meets the recommended level, while methionine + cystine (~0.36–0.38 g/100 g protein), although relatively low, meets the total sulfur amino acid requirement. These findings indicate that mealworms are a high-biological-value protein source with a balanced essential amino acid profile. Millward (57) emphasizes that leucine, isoleucine, lysine, and threonine are critical for protein quality. Furthermore, Iaconisi et al. (58) reported that mealworm flour significantly altered the amino acid composition in fish feed.

Among literature, *T. molitor* L. has positive findings on AA profile and high nutritional quality. Ghosh et al. (59) detected up to 17 amino acids in mealworm larvae from commercial production, and reported that the content of leucine, valine, isoleucine and lysine in the samples especially satisfied FAO/WHO/UNU (56) recommendations. Despite their low methionine level, mealworms are a great source of protein for biological purposes due to their abundance of the other essential amino acids. Similarly, Da Silva et al. (60) noted that mealworm flour is highly proteinaceous and lipid-rich, containing essential amino acids like histidine, lysine, and valine with variability depending on feed treatments. These results suggest that mealworms are nutritionally beneficial due to their high protein and essential amino acid content; however, the exact amino acid profile may vary depending on the additives and diet they are fed. We also found that mealworm larvae given turmeric exhibited alterations in their amino acid composition and outcomes comparable to those reported in the literature. Meyer-Rochow et al. (53) found mealworm species to be high in glutamic acid as well, with substantial levels of leucine and lysine among the essential amino acids. The reason that all of these studies show mealworms being a great source for essential aminoacids (EAA) and umami is probably the similarity, which can notice between them. This variation in amino acid profile observed for mealworm larvae samples fed with turmeric in present study may be the reason of these high nutritive values reported elsewhere and indicates that the substrate diet can greatly influence the composition of amino acids. After analyzing at all of the essential amino acid levels in the mealworms, we found that the group that had turmeric (Tm-T) had a total of 6.00 g/100 g.

Generally, *T. molitor* larvae fed turmeric exhibited improved amino acid profiles, including higher glutamic acid, aspartic acid, serine, threonine, and tyrosine levels. This enrichment achieved a balanced essential amino acid profile, excellent biological value, and enhanced functional quality. The amino acid profile of turmeric-fed mealworms meets FAO/WHO/UNU (Food and Agriculture Organization/World Health Organization/United Nations University) reference requirements for adult nutrition, indicating their potential as a high-quality protein source.

### 3.5 Fatty acid composition

The fatty acid values for the *T. molitor* larvae and turmeric samples in the Tm-C, Tm-T, and T groups are presented in Table 4. All groups exhibited varying concentrations of *γ*-linolenic acid (C18:3n6), linoleic acid (C18:2c), arachidonic acid (C20:4), palmitic acid (C16:0), myristic acid (C14:0), oleic acid (C18:1c), nervonic acid (C24:1), *α*-linolenic acid (C18:3n3), behenic acid (C22:0), lignoceric acid (C24:0), and stearic acid (C18:0). Certain fatty acids (e.g., butyric, caproic, caprylic, lauric, undecanoic) were absent in both the control and experimental groups across all sample groups, although were present in significant quantities in turmeric root.

**Table 4:** Fatty acid composition (%) and one-way ANOVA results for the three samples (Tm-C, Tm-T, T).

Fatty acid/Group (%)	Tm-C (%)	Tm-T (%)	T (%)
α-Linolenic acid (C18:3n3)	0.71 ± 0.01^a^	0.65 ± 0.01^a^	1.07 ± 0.04^c^
Caprylic acid (C8:0)	nd	nd	1.60 ± 0.02^a^
Undecanoic acid (C11:0)	nd	nd	2.32 ± 0.16^a^
Lauric acid (C12:0)	nd	nd	0.30 ± 0.04^a^
Tridecanoic acid (C13:0)	nd	nd	0.66 ± 0.01^a^
Myristic acid (C14:0)	2.78 ± 0.01^a^	2.27 ± 0.01^a^	0.28 ± 0.01^b^
Palmitic acid (C16:0)	17.34 ± 0.02^a^	11.42 ± 0.01^b^	1.08 ± 0.01^c^
Oleic acid (C18:1c)	44.41 ± 0.05^a^	28.92 ± 0.03b	17.97 ± 0.07^c^
Butyric acid (C4:0)	nd	nd	3.57 ± 0.62^a^
Linoleic acid (C18:2c)	23.95 ± 0.03^a^	16.59 ± 0.01^b^	1.91 ± 0.27^c^
Heptadecenoic acid (C17:1)	1.09 ± 0.01^c^	8.66 ± 0.01^b^	35.27 ± 0.47^a^
Palmitoleic acid (C16:1)	2.41 ± 0.01^a^	1.92 ± 0.01b	0.35 ± 0.05^c^
Lignoceric acid (C24:0)	0.43 ± 0.01^b^	0.66 ± 0.01a	nd
Caproic acid (C6:0)	Nd	nd	9.35 ± 0.21^a^
γ-Linolenic acid (C18:3n6)	1.25 ± 0.01^b^	10.18 ± 0.01^a^	0.17 ± 0.01^c^
Capric acid (C10:0)	Nd	nd	2.28 ± 0.05^a^
Arachidonic acid (C20:4)	0.49 ± 0.01^b^	1.22 ± 0.01^a^	nd
Behenic acid (C22:0)	1.97 ± 0.01^a^	nd	nd
Stearic acid (C18:0)	2.35 ± 0.01^a^	1.55 ± 0.01^b^	0.61 ± 0.07^c^
Nervonic acid (C24:1)	nd	2.12 ± 0.0^1a^	0.10 ± 0.01^b^
Saturated fatty acids (SFA)	24.99 ± 0.02	18.96 ± 0.01	23.50 ± 0.49
Monounsaturated FA (MFA)	47.91 ± 0.06	41.62 ± 0.02	70.96 ± 0.67
Polyunsaturated FA (PUFA)	26.41 ± 0.03	39.08 ± 0.02	5.22 ± 0.36

Tm-C, Mealworm control; Tm-T, Mealworm fed with turmeric; T, Turmeric root; n.d., not determined. The data are expressed as the mean ± standard deviation (*n* = 3). When comparing the means of different letters in a given row (a–c), a significant difference between the groups was indicated by the Tukey HSD test (*p* < 0.05). ANOVA, one-way analysis of variance; SD, standard deviation.

In terms of total fatty acid level, from high to low; monounsaturated fatty acids (%47.91 ± 0.06) were predominant in the Tm-C group, polyunsaturated fatty acids (%39.08 ± 0.02) in the Tm-T group, and monounsaturated fatty acids (%70.96 ± 0.67) in the T group. Compared to the Tm-C group, a decrease in oleic acid levels (44.41% → 28.92%) and linoleic acid levels (23.95% → 16.59%) was observed in the Tm-T group, while a significant increase was observed in γ-linolenic acid levels (1.25% → 10. 18) and heptadecenoic acid (1.09% → 8.66%) levels were significantly increased (*p* < 0.05). In the T group, heptadecenoic acid (35.27%) and trans-elaidic acid (16.50%) were found at the highest levels, indicating that turmeric’s unique fatty acid profile is also reflected in the larvae.

The fatty acid detected at the highest level in all sample groups was oleic acid (C18:1c), while heptadecenoic acid (C17:1) was identified as the predominant fatty acid in the T group. The fatty acids detected at the lowest levels in the Tm-C and Tm-T groups were behenic and lignoseric acids, while nervonic acid was found at the lowest level in the T group.

The fatty acids detected at the highest levels in the control group were oleic acid (C18:1c) (44.41%) and linoleic acid (C18:2c). In the group fed turmeric, the oleic acid content decreased to 28.92%, while linoleic acid (C18:2c, 18-carbon polyunsaturated fatty acid) decreased from 23.95 to 16.59%. In contrast, gamma-linolenic acid (C18:3n6, polyunsaturated fatty acid) showed the most significant increase, rising from 1.25% in the control group to 10.18% in the turmeric group. Compared with the control sample, turmeric feeding increased *γ*-linolenic acid by approximately 8-fold, but oleic acid remained the predominant fatty acid. This indicates that turmeric particularly enriches the PUFA profile. Paul et al. (61) found that oil obtained from fresh turmeric (*C. longa* L.) rhizomes grown in Bangladesh contained 8.76–10.92% fat and that the fatty acid composition had the highest proportion of oleic acid (C18:1, 56. 24–58.88%); followed by Myristic Acid (C14:0, 16.25–17.71%), Palmitic Acid (C16:0, 5.59–6.00%), Linoleic Acid (C18:2, 10.90–12.82%), Linolenic Acid (C18:3, 4.15–5.46%), and Eicosenoic Acid (C20:1, 2.72–3.25%).

In this study, the fatty acid profile of mealworm larvae fed with turmeric changed qualitatively, with some specific components such as γ-linolenic and heptadecenoic acids increasing, while the ratios of oleic and linoleic acids decreased. Similarly, Copelotti et al. (62) reported in their study that the fatty acid composition of these mealworm larvae varied significantly depending on their feeding environment. For this purpose, six different diets were prepared by mixing wheat bran (control) with fully hydrogenated rapeseed oil rich in stearic acid (C18:0, 91%) at different ratios, and the larvae were fed for 2 weeks. It was reported that diets rich in saturated fatty acids increased the total lipid content in the larvae; while polyunsaturated fatty acid (PUFA) levels decreased, C18:0 and saturated fatty acids (SFA) increased significantly. These findings demonstrate that, thanks to the metabolic flexibility of mealworms, their fatty acid profiles can be directed through nutrition, and that the substrate is a critical factor in obtaining food or feed products with different functional properties.

Dreassi et al. (63) demonstrated that the fatty acid composition of *T. molitor* larvae is sensitive to the dietary substrate and that different diets significantly alter the fatty acid profile without changing the total fat content. In particular, diets based on bread and oat flour were noted to yield a more favorable n-6/n-3 ratio, whilst the combination of brewer’s yeast, wheat, and oats provided both a superior lipid profile and commendable growth performance. Similarly, Lawal et al. (64) reported that oilseed supplements improved the profile by increasing omega-3 levels. These findings are consistent with turmeric directing the mealworm fatty acid composition in our study. These findings confirm that the fatty acid profiles of mealworms can be directed through their diet, thereby increasing their potential for use as functional foods.

Fatty acids (FA) are essential constituents of cell membranes and exert considerable influence on cardiovascular health. In particular, polyunsaturated fatty acids (PUFAs) stand out for their antiatherogenic properties, while oleic and linoleic acids contribute to energy metabolism (65, 66). Our findings indicate that increased PUFA levels in the Tm-T group enhance the functional food potential of mealworms. It is known that reducing SFA intake and increasing PUFA consumption lowers the risk of coronary heart disease. Furthermore, the mealworm’s rich n-6 and n-3 PUFAs offer a profile that may provide cardiovascular benefits, as reported in the literature (63).

### 3.6 Mineral composition and safety assessment

Turmeric root (T) exhibited a high concentration of macroelements, particularly K (7097.12 ± 91.57 ppm) and Ca (760.84 ± 4.83 ppm), while the levels of these elements were lower in larvae (K: 3392.52 ± 23.23–3884.01 ± 47.67 ppm; Ca: 164.17 ± 3.05–168.32 ± 3.80 ppm; *p* < 0.05). Although feeding with turmeric increased the K level (Tm-C: 3392.52 ± 23.23 ppm → Tm-T: 3884.01 ± 47.67 ppm), it did not approach the level in the plant matrix; Ca remained at similar levels in both larval groups (*p* < 0.05). In contrast, Na (515.07 ± 4.67 → 553.31 ± 7.32 ppm), Mg (938.22 ± 5.36 → 1272.90 ± 13.06 ppm) and P (3008.61 ± 15.58 → 3657.40 ± 43.78 ppm) contents were significantly increased in Tm-T (*p* < 0.05) (Table 5).

**Table 5:** Mineral concentrations (ppm) and one-way ANOVA results for three samples (Tm-C, Tm-T, T).

Element	Tm-C (ppm)	Tm-T (ppm)	T (ppm)
Na	515.07 ± 4.67^b^	553.31 ± 7.32^a^	21.47 ± 0.14^c^
K	3392.52 ± 23.23^b^	3884.01 ± 47.67^c^	7097.12 ± 91.57^a^
Ca	164.17 ± 3.05^b^	168.32 ± 3.80^b^	760.84 ± 4.83^a^
Mg	938.22 ± 5.36^b^	1272.90 ± 13.06^a^	745.88 ± 12.63^c^
P	3008.61 ± 15.58^b^	3657.40 ± 43.78^a^	1242.82 ± 5.79^c^
B	n.d	n.d	n.d
Co	0.00 ± 0.00^b^	0.00 ± 0.00^b^	0.04 ± 0.00^a^
Cr	0.67 ± 0.06^a^	0.69 ± 0.03^a^	0.11 ± 0.01^b^
Cu	8.05 ± 0.05^a^	8.12 ± 0.10^a^	1.72 ± 0.09^b^
Fe	19.05 ± 0.46^a^	19.72 ± 0.14^a^	13.12 ± 0.42^b^
Li	0.16 ± 0.02^a^	0.08 ± 0.01^b^	0.05 ± 0.01^c^
Mn	4.11 ± 0.16^b^	5.09 ± 0.12^c^	14.26 ± 0.26^a^
Mo	0.35 ± 0.02^b^	0.38 ± 0.03^b^	0.81 ± 0.02^a^
Ni	0.03 ± 0.00^b^	0.17 ± 0.01^a^	0.14 ± 0.01^c^
Se	0.13 ± 0.02^a^	0.11 ± 0.04^a^	0.01 ± 0.01^b^
Si	13.18 ± 0.36^b^	17.93 ± 0.14^c^	72.62 ± 2.03^a^
Zn	43.61 ± 0.57^b^	61.11 ± 0.13^a^	4.09 ± 0.37^c^
Pb	0.06 ± 0.01^b^	0.03 ± 0.00^c^	0.09 ± 0.00^a^
Cd	0.05 ± 0.00^a^	0.06 ± 0.00^a^	0.03 ± 0.00^b^
Hg	0.03 ± 0.00^b^	0.02 ± 0.00^b^	0.04 ± 0.00^a^
As	n.d	n.d	n.d
Al	0.97 ± 0.24^b^	0.97 ± 0.09^b^	13.58 ± 0.47^a^
Sb	0.07 ± 0.00^a^	0.07 ± 0.00^b^	0.05 ± 0.00^c^
Be	0.01 ± 0.00^ab^	0.01 ± 0.00^b^	0.01 ± 0.00^a^
Ag	0.02 ± 0.00^a^	0.01 ± 0.00^b^	0.01 ± 0.00^c^

Tm-C, Mealworm control; Tm-T, Mealworm fed with turmeric; T, Turmeric root; n.d., not determined. Values are given as mean ± SD (*n* = 3). When comparing the means of different letters in a given row (a–c), a significant difference between the groups was indicated by the Tukey HSD test (*p* < 0.05). ANOVA, one-way analysis of variance; SD, standard deviation.

The increase in Mg and P suggests that turmeric supplementation did not uniformly increase all macrominerals; it elicited a selective response via element-specific bioavailability and/or intestinal retention. In particular, the finding that Mg was 745.88 ± 12.63 ppm in the T sample but 1272.90 ± 13.06 ppm in Tm-T indicates that mineral homeostasis in larvae cannot be explained solely by feed content, suggesting that physiological regulation may play a dominant role. Similarly, the increase in P (≈ + 649 ppm) indicates that the feeding intervention may enhance nutritional quality, given phosphorus’s role in energy metabolism and phospholipid structure (67, 68).

The most significant change among trace elements was observed for Zn, increasing from 43.61 ± 0.57 ppm to 61.11 ± 0.13 ppm in Tm-T (p < 0.05); considering this increase together with the low Zn level in the T sample (4.09 ± 0.37 ppm), it strengthens the possibility of modulation in uptake/retention mechanisms rather than direct “Zn transfer in the matrix”. In addition, Mn (4.11 ± 0.16 → 5.09 ± 0.12 ppm) and Si (13.18 ± 0.36 → 17.93 ± 0.14 ppm) increased; while Ni increased from 0.03 ± 0.00 ppm to 0.17 ± 0.01 ppm (p < 0.05). In contrast, Fe (≈19–20 ppm), Cu (≈8 ppm), and Cr (≈0.67–0.69 ppm) were conserved among larval groups; patterns such as the decrease in Li (0.16 ± 0.02 → 0.08 ± 0.01 ppm) and the undetectable Co in larvae (0.00 ppm) support the idea that mineral composition does not linearly reflect the plant matrix and that there is element-specific physiological regulation (67, 68).

In terms of potentially toxic elements, turmeric supplementation was not associated with adverse accumulation: Pb decreased from 0.06 ± 0.01 ppm in Tm-C to 0.03 ± 0.00 ppm in Tm-T, Hg remained in the range of 0.02–0.03 ppm, and As was undetectable in any sample (n.d.). Cd levels ranged from 0.05 to 0.06 ppm and did not show a significant increase in larvae. The high Al content in the T sample (13.58 ± 0.47 ppm) and its low, constant level in larvae (0.97 ± 0.24 and 0.97 ± 0.09 ppm) also indicate that metal uptake is limited by insect physiology. Similarly, the very low levels observed for trace metals such as Sb, Be, and Ag support the overall safety profile under the conditions studied.

### 3.7 Multivariate pattern recognition and clustering analysis

**Figure 3:**
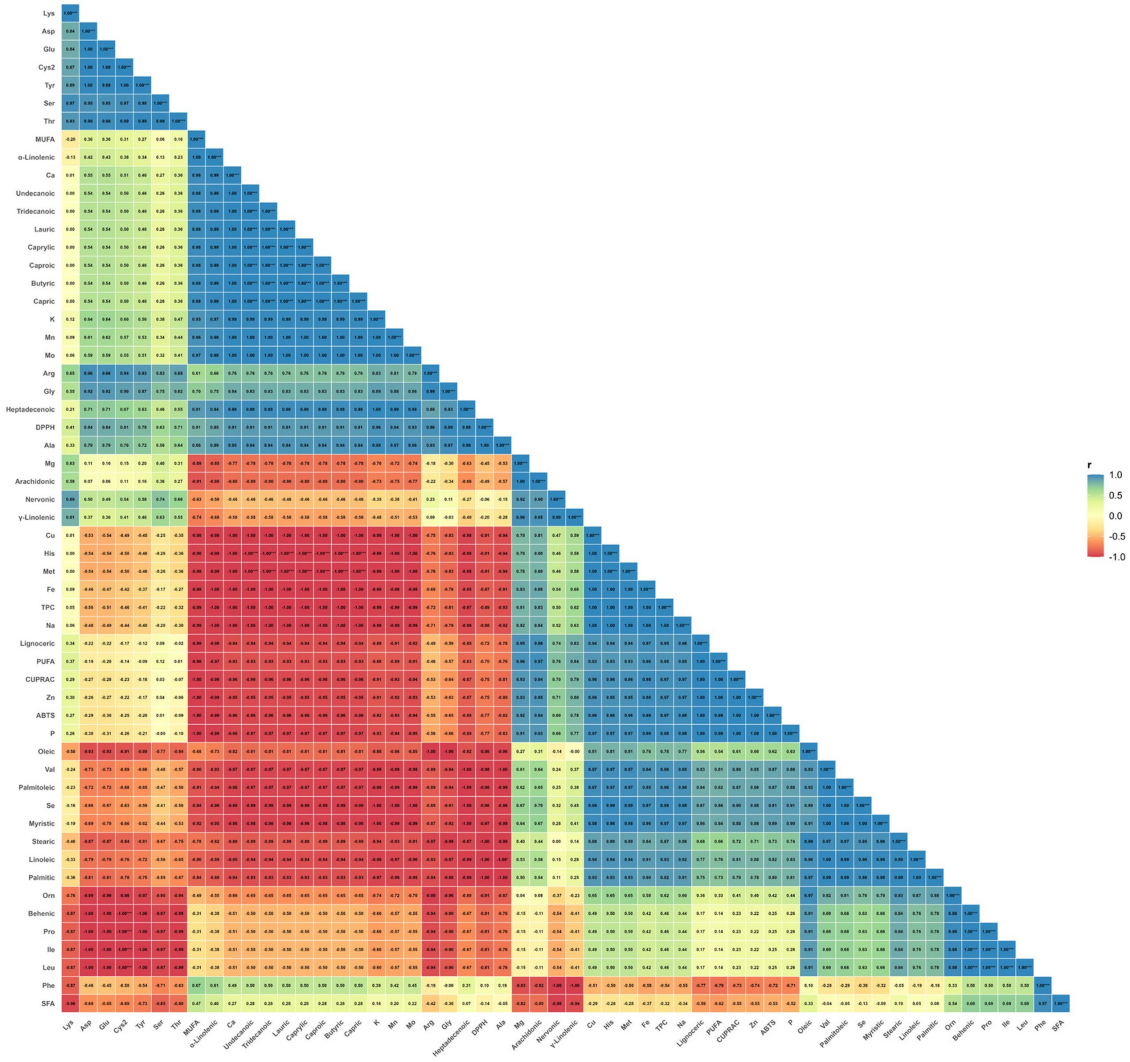
Pearson correlation heatmap showing pairwise associations among total phenolic content (TPC), antioxidant capacity indices, amino acid composition, mineral profile (excluding heavy metals), and fatty acid composition across the analyzed samples: T (turmeric root), Tm-C (mealworm control), and Tm-T (mealworm fed with turmeric). Cells report Pearson’s correlation coefficients (*r*), and the color scale indicates correlation direction and magnitude (−1 to +1). *p*-values were adjusted using the Benjamini–Hochberg procedure; given the limited number of observations (*n* = 3), statistical significance should be interpreted with caution and used primarily for exploratory inference.

Figure 3 shows that the variables exhibit a block structure in the integrated dataset obtained from samples T (turmeric root), Tm-C (mealworm control), and Tm-T (mealworm fed with turmeric). Within the antioxidant axis, an almost perfect positive correlation is observed between ABTS and CUPRAC (r = 0.9998); similarly, the strong positive correlation of TPC with both ABTS (r = 0.9737) and CUPRAC (r = 0.9696) suggests that the phenolic pool acts in conjunction with the total reducing capacity, particularly based on ABTS/CUPRAC. In contrast, the inverse correlation of DPPH with TPC (r = −0.8923) and its negative relationship with ABTS/CUPRAC (r ≈ −0.77 to −0.75) indicate that DPPH exhibits a different dynamic in this dataset (e.g., matrix- or composition-related sensitivity of the radical-scavenging mechanism). While these correlation patterns are noteworthy, the interpretation of the findings should be based on the overall structure and clustering tendencies formed by the correlations together, rather than on individual coefficients. Another quantitative finding that stands out in Figure 3 is that some minerals are located in the same block as the antioxidant indices. For example, the very strong positive relationship of Zn with ABTS (r = 0.9996) and CUPRAC (r = 0.9999) shows that in this dataset, the Zn level tends to increase along with antioxidant capacity; Indeed, the amount of Zn is 61.11 in Tm-T, 43.61 in Tm-C, and 4.09 in T. Similarly, the correlation of P with ABTS (r = 0.9999) and the correlation of Na with TPC (r = 0.9998) support a clustering in the same direction. In contrast, the significant negative relationship of Ca with TPC (r = −0.9986) is a parallel pattern to the fact that Ca is very high in sample T with 760.84, while TPC is lower in T with 730.22 (Ca ~ 164–168 in Tm-C and Tm-T, while TPC ~ 974–987). At this point, it should be emphasized that the correlations do not claim “causality”; rather, they indicate clusters of parameters that vary together between samples.

**Figure 4:**
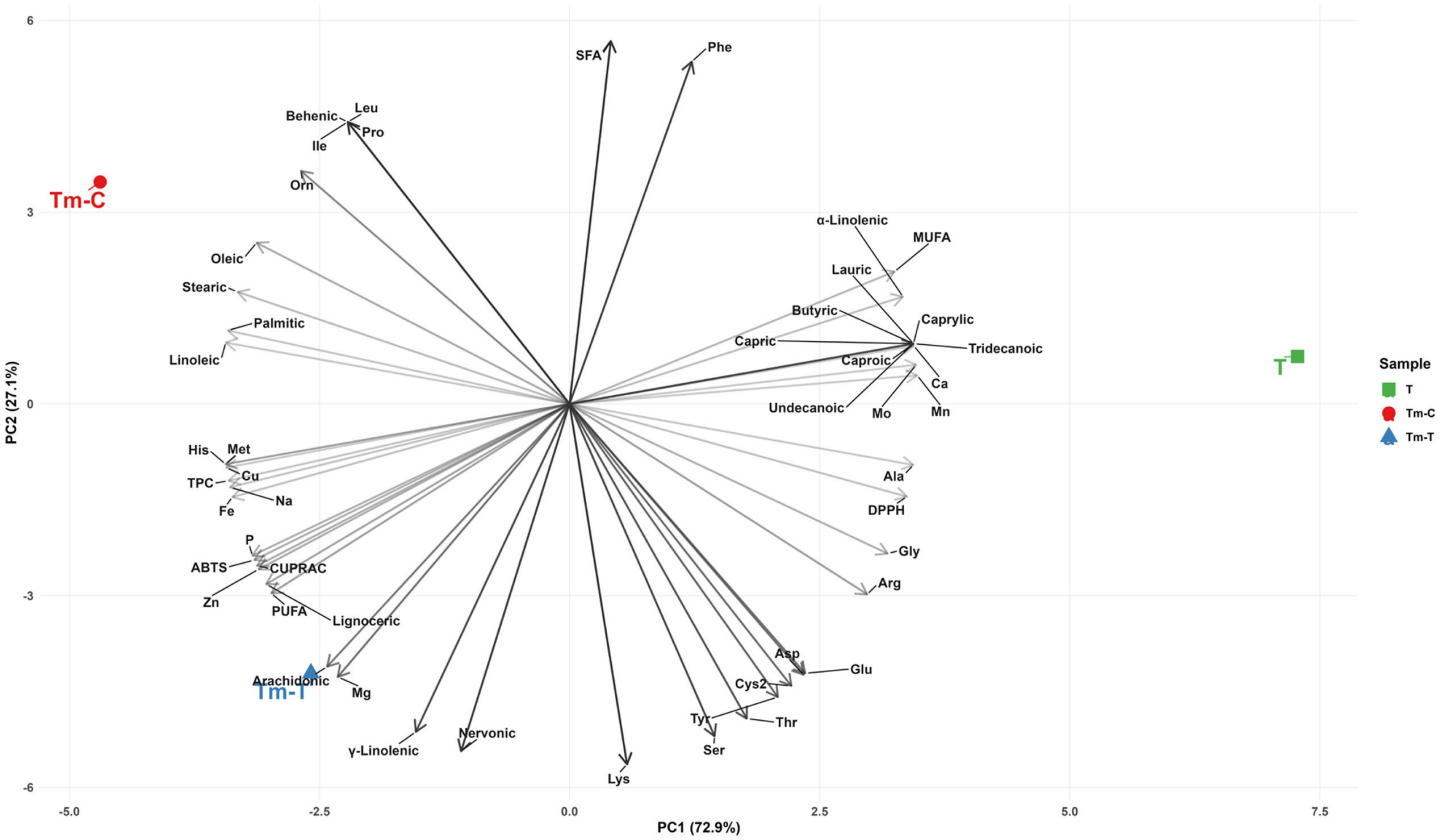
Principal component analysis (PCA) of the integrated compositional dataset obtained from T (turmeric root), Tm-C (mealworm control), and Tm-T (mealworm fed with turmeric) samples. Prior to PCA, variables were centered and scaled (*z*-score standardization) to ensure comparable weighting across measurement scales.

Figure 4 (PCA) quantitatively summarizes how these correlation blocks separate the samples. PC1 explains 70.9% of the total variance, and PC2 explains 29.1% (since there are 3 samples, the first two components together account for 100%). The samples are clearly separated along the PC1–PC2 plane. The score values show that sample T is separated from the two larval samples by being positioned positively in PC1 (approximately +8.57); Tm-C is more in the PC1 negative–PC2 negative region (approximately −5.84, −4.18), while Tm-T is separated positively in PC2 (approximately −2.73, +5.33). This separation suggests that, in practice, the principal axis of “T matrix ↔ larval matrix” is captured by PC1; and within the larvae, the difference of “control ↔ turmeric feeding” is carried to PC2. When the “visible drivers” of this separation in Figure 4 are examined numerically, it is seen that the components favoring Tm-T in the PC2 direction are particularly prominent in the lipid fraction: PUFA is at 39.08 in Tm-T, exceeding Tm-C’s value of 26.41, while PUFA is at 5.22 in the T sample. One of the most striking differences is seen in *γ*-linolenic acid: Tm-T 10.18, Tm-C 1.25, T 0.17. In parallel, the observation of SFA at 18.96 in Tm-T, lower than in Tm-C (24.99) and T (23.50) samples, supports the idea that the separation in PC2 fits along the “higher PUFA/γ-linolenic acid, lower SFA” axis. On the antioxidant side, while Tm-T’s ABTS (45.56) and CUPRAC (1192.92) values are higher compared to Tm-C (ABTS 39.22, CUPRAC 969.14), T’s ABTS (23.00) and CUPRAC (439.86) remain lower; this table is consistent with the positioning in PCA.

**Figure 5:**
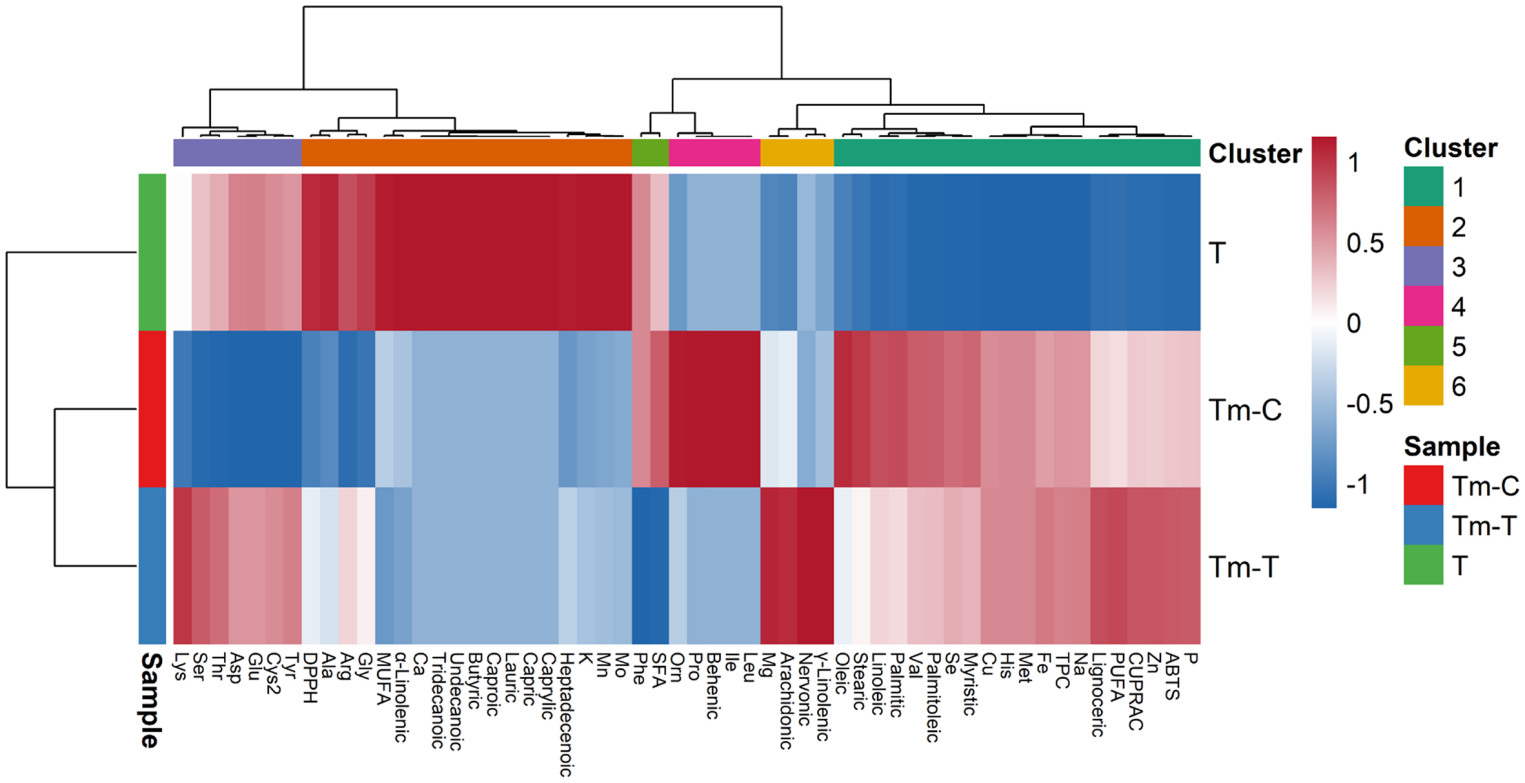
Hierarchically clustered heatmap of *z*-score–standardized variables across T (turmeric root), Tm-C (mealworm control), and Tm-T (mealworm fed with turmeric) samples, generated using Euclidean distance and Ward.D2 linkage. The top annotation bar indicates feature cluster membership (*k* = 6), while the row annotation denotes sample identity. Heatmap intensities reflect relative increases or decreases (*z*-scores) of each variable across samples, highlighting co-varying feature modules.

Figure 5 (HCA clustered heat map after *z*-score standardization) makes the PCA-derived separation visible at the module level and clarifies that the variables are organized into clusters that show covariation. When viewed through the colored upper annotation (*k* = 6), the clustering of antioxidant/phenolic indicators such as TPC–ABTS–CUPRAC in the same module as minerals such as Zn, P, Na, and some fatty acids shows that there is a bidirectional pattern among “antioxidant-mineral-lipid components” across the samples. In contrast, the positioning of DPPH in a different module reinforces the idea that DPPH is decoupled from the ABTS/CUPRAC/TPC axis in this dataset, consistent with the negative correlations in Figure 3. Furthermore, in the same heat map, the formation of “hot blocks” of lipid markers such as PUFA/*γ*-linolenic acid, which are significantly elevated in Tm-T, and the different density profile of minerals such as Ca and K (e.g., K: 7097.12, Ca: 760.84) in the T sample, provide visual evidence that enhances the biochemical significance of the clusters.

In conclusion, when the three figures are evaluated together, it is seen that: (i) ABTS and CUPRAC, representing antioxidant capacity, change in the same direction as total phenolic content (TPC) and the relationship between them is strong (r ≈ 0.97), (ii) in PCA, PC1 is the dominant component explaining the main divergence between samples (70.9%) and PC2 reflects the change in lipid fraction, especially in the Tm-T sample (increase in PUFA/γ-linolenic acid and decrease in SFA), (iii) HCA visualizes this divergence through module/cluster structures, highlighting the groups of variables that change together. The consistent emergence of divergence in this triple set of evidence indicates that the difference between samples is not due to a single parameter; The phenolic-antioxidant responses are shown to be fueled by a “compositional signature” change resulting from the co-rearrangement of mineral and fatty acid composition, and the Tm-T sample in particular exhibits a distinctive biochemical orientation characterized by a higher unsaturation profile (increase in PUFA/γ-linolenic acid).

## 4 Conclusion

Turmeric-supplemented feeding can be considered an effective “nutritional steering” strategy for directing the nutritional composition of mealworm larvae, and can provide selective enrichment, particularly along the Mg–P–Zn axis. However, the limited accumulation of certain minerals, such as Ca and K, indicates the need for additional formulation strategies to achieve targeted mineral fortification. Since batch-dependent trace-element variability has been reported in commercial turmeric products, batch-based contaminant monitoring and standardized metadata reporting of turmeric growing/feeding conditions are recommended for scale-up studies. Evaluating the bioavailability of mineral fractions through validation studies with different turmeric sources and feeding durations will strengthen the translation of the findings into functional food/feed claims. In the study as a whole, it was determined that fat and carbohydrates decreased, and protein increased, in samples fed turmeric; furthermore, the high proportion of glutamic acid provided an important clue for product development regarding umami contribution. The increase in total phenolic content and antioxidant activity is thought to stem from turmeric’s rich phenolic profile and strong antioxidant capacity. Applying similar approaches to other edible insect species has the potential to increase bioactive content and bioavailability.

## Data Availability

The raw data supporting the conclusions of this article will be made available by the authors, without undue reservation.
