# Supplementary material for: Determination of nutritional values of mealworm (Tenebrio molitor L.) larvae fed with turmeric (novel food product)
Source: Front Nutr. 2026 Apr 14;13:1812226. doi: 10.3389/fnut.2026.1812226 (PMC13120943; doi:10.3389/fnut.2026.1812226)
Supplement: Supplementary file 1 [file Supplementary_file_1.docx]

**Supplemantary Files**

**Determination of Nutritional Values ​​of Mealworm (*Tenebrio molitor* L.) Larvae Fed with Turmeric (Novel Food Product)**

Deniz Aktaran Bala^1*^, Sema Sandıkçı Altunatmaz^1^, Filiz Aksu^1^, Cansu Çelik Doğan^1^, İbrahim Akyazı^2^, Dilek Dülger Altıner^3^, Emine Aydın^4^**,** Seydi Yıkmış^5*^, Emad Karrar^6^, Moneera O. Aljobair^7*^_,_ Isam A. Mohamed Ahmed^8^

^1^Department of Food Processing, Vocational School of Veterinary Medicine, Istanbul University-Cerrahpasa, Avcilar, Istanbul, Türkiye

^2^Department of Physiology, Faculty of Veterinary Medicine, Istanbul University-Cerrahpasa, Avcilar, Istanbul, Türkiye

^3^Department of Gastronomy and Culinary Arts, Tourism Faculty, Kocaeli University, Kocaeli, Türkiye

^4^Department of Agricultural Biotechnology, Faculty of Agriculture, Düzce University, Düzce, Türkiye

^5^Department of Food Technology, Tekirdag Namik Kemal University, 59830 Tekirdag, Türkiye

^6^Department of Plant Sciences, North Dakota State University, Fargo 58108 ND, USA ^7^Department of Sports Health, College of Sports Sciences and Physical Activity, Princess Nourah bint Abdulrahman University, Riyadh, Saudi Arabia

^8^Department of Food Sciences and Nutrition, College of Food and Agricultural Sciences, King Saud University, P. O. Box 2460, Riyadh 11451, Saudi Arabia

***Corresponding author E-mail:** [syikmis@nku.edu.tr](mailto:syikmis@nku.edu.tr), [deniz.bala@iuc.edu.tr](mailto:deniz.bala@iuc.edu.tr), moaljobair@pnu.edu.sa


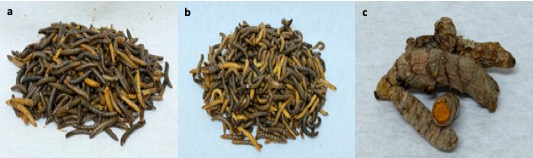


**Figure S1. a.** Control (Tm-C) **b.** Turmeric-fed group (Tm-T) **c.** Turmeric (T)

**Figure S2.** Calibration curve for total phenolic content

**Figure S3.** Antioxidant activity calibration graph of free and bound phenols according to the ABTS method

**Fig S4.** Antioxidant activity calibration graph of free and bound phenols according to the CUPRAC method

**Fig S5.** Antioxidant activity calibration graph of free and bound phenols according to the DPPH method

**Table S1.** GC–FID operating conditions

| **Parameter** | **Condition** |
| --- | --- |
| **Instrument** | Agilent 6890N Gas Chromatograph with ECD/FID detector and Split/Splitless inlet |
| **Column** | HP‑88, 100 m × 0.25 mm × 0.20 µm |
| **Oven Temperature Program** | 120 °C for 1 min; ramp at 10 °C/min to 175 °C, hold 10 min; ramp at 3 °C/min to 210 °C, hold 5 min; ramp at 5 °C/min to 240 °C, hold 5 min |
| **Injector Temperature** | 250 °C |
| **Detector Temperature** | 280 °C |
| **Injection Volume** | 1 µL |
| **Carrier Gas** | Helium, 2 mL/min |
